# Clonal Complex 398 Methicillin Susceptible *Staphylococcus aureus*: A Frequent Unspecialized Human Pathogen with Specific Phenotypic and Genotypic Characteristics

**DOI:** 10.1371/journal.pone.0068462

**Published:** 2013-11-15

**Authors:** Tomasz Chroboczek, Sandrine Boisset, Jean-Philippe Rasigade, Anne Tristan, Michele Bes, Helene Meugnier, François Vandenesch, Jerome Etienne, Frederic Laurent

**Affiliations:** 1 French National Reference Centre for Staphylococci, Hospices Civils de Lyon, Lyon, France; 2 Réanimation médicale - Pavillon N, Hopital Edouard Herriot, Hospices civils de Lyon, Lyon, France; 3 Laboratoire de Bactériologie - Département des Agents Infectieux, Centre Hospitalier Universitaire de Grenoble, Grenoble, France; 4 Centre National de la Recherche Scientifique – Unité Mixte de Recherche 5163, Université Joseph Fourier, Grenoble, France; 5 University of Lyon – Institut National de la Santé et de la Recherche Médicale Unité 851, Faculté de Médecine Lyon Est, Lyon, France; University of Iowa, United States of America

## Abstract

Clonal complex 398 livestok-associated-MRSA (CC398 LA-MRSA) clone is described as a major animal pathogen that can also colonize and infect humans. CC398 methicillin susceptible *Staphylococcus aureus* (CC398 MSSA) is less described. We identified 126 CC398 MSSA strains of human origin within 6380 *S. aureus* isolates gathered between 2009 and 2011, from the French National Reference Centre for Staphylococci. They were characterized using antimicrobial susceptibility testing, *spa* typing, DNA microarrays (Identibac *S. aureus* Genotyping ®, Alere), CC398-specific sequence PCR, *erm*T (encoding macrolides résistance) PCR. Fifty-three CC398 LA-MRSA collected from French pigs and veal were used as comparators, and phylogenetic relations between human CC398 MSSA and animal CC398 MRSA populations were explored on the basis of *spa*-typing and DNA microarrays. CC398 MSSA were able to induce a large spectrum of infections (especially skin, bloodstream, and pneumonias). The prevalence rate of this clone was high in MSSA population, i.e., 24.7% in a local prospective study on nasal colonization, and 7.5% in a national prospective study on infective endocarditis. CC398 MSSA isolates were frequently (89%) erythromycin resistant, due to the presence of the *erm*T gene, a gene not detected in erythromycin resistant CC398 LA-MRSA strains. Expression of staphylococcal complement inhibitor (*scn*) and the chemotaxis inhibitory protein (*chp)*, was also specific to this population. The CC398 MRSA signature included also a panel of antibiotic resistance genes, especially a type IV or V cassette *mec* and *tetM*. CC398 MSSA and CC398 LA-MRSA populations were closely related based on *spa*-typing and DNA microarrays, with the MRSA strains forming the most derived lineage in phylogenic trees. Both MSSA and MRSA populations may come from common ancestors, which would have evolved in the settings of different selective pressures, explaining the acquisition of *ermT*, *chp* and *scn* for MSSA, and antibiotic resistance genes for MRSA.

## Introduction

*Staphylococcus aureus* is both a human commensal organism and a pathogen [1] and inter-human transmission is the main route of its dissemination. Wild strains are susceptible to methicillin (MSSA), and methicillin resistance acquisition can be healthcare associated (HA-MRSA), or community associated (CA-MRSA) [2], [3]. Since the 2000s, livestock animals, especially pigs, have been described to be a third reservoir of MRSA (called livestock-associated MRSA (LA-MRSA)). Particularly, MRSA belonging to sequence type 398 (ST398) or related, clustering in clonal complex 398 (CC398), have emerged world-wide and have been described to be able to colonize a large panel of farm animals. The transmission of these MRSA to humans, leading to colonization but also to infection, has been now reported in many countries [4], [5].

MRSA clones usually emerge from corresponding reservoir of MSSA by acquisition of the *SCCmec* element, as described for the epidemic CA-MRSA ST8 (USA300) clone [6], [7] and the European CA-MRSA ST80 clone [1]. While ST398 MRSA is mainly detected in animals, the corresponding ST398 MSSA has been less reported in animals [8], [9]. Conversely, several reports of ST398 MSSA in humans have been recently made. A low prevalence of such strains among *S. aureus* colonizing isolates was reported in the Netherlands in 2008 (2/829, i.e 0.2%) [9] or in Spain in 2011 (2/52, i.e. 3.7%) [10]. Recently, Baht *et al.* highlighted that in Northern Manhattan (New York, USA), 13 out of 914 screened people (1,4%) were found to be colonized by ST398 MSSA isolates. In the same way, cases of infection due to CC398 MSSA have been reported, in France (bloodstream infection, n = 18 [11]; and necrotizing pneumonia, n = 1 [12]), in Colombia (bloodstream infection, n = 1 [13]) or in Belgium (bloodstream infection, n = 1; respiratory tract infection, n = 2; and wound infection, n = 2) [14]. Finally, in China, Dominican Republic and Martinique, prevalence of ST398 within MSSA collected both from colonization and infection reached 18.9% (31/164) [15], 7.8% (7/90) [16] and 10.4% (9/87) [16], respectively, without details on distribution of infection and colonization cases in each study.

If animal and human CC398 MRSA strains have been extensively explored, their human MSSA counterparts have been poorly studied. Herein, we investigated clinical, phenotypic, genotypic features of the largest collection of such isolates collected in France, country where MRSA CC398 have been initially described in 2005 [17] and where emergence of CC398 MSSA have been first reported [18]. Molecular data were compared to those obtained from French CC398 LA-MRSA isolates.

## Results

**Figure 1:**
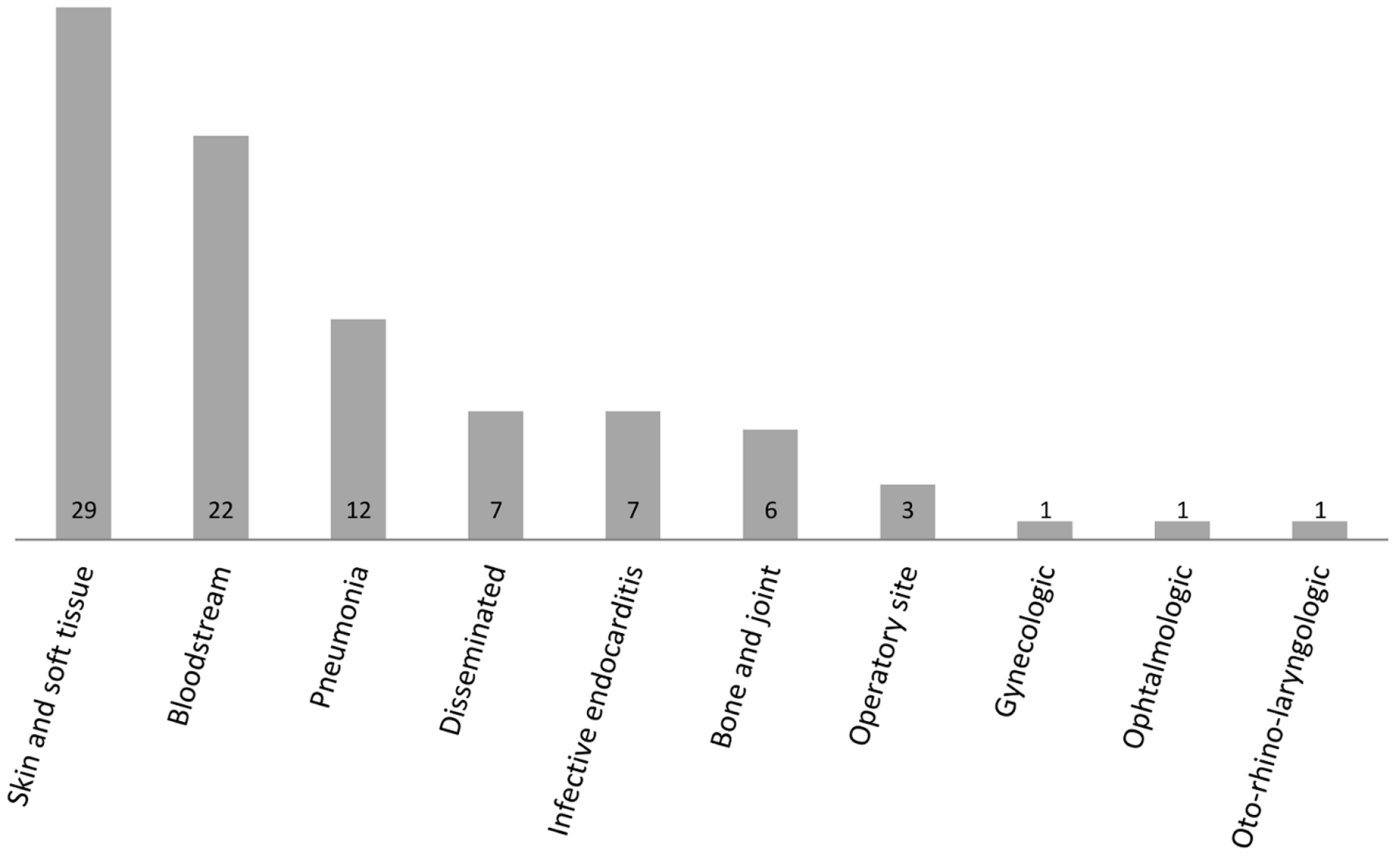
Nature and number of infections caused by 89 MSSA CC398 strains collected in mainland France between 1999 and 2011. Disseminated infections are defined by the presence of septic metastasis in at least 2 noncontiguous organs (by opposition to bloodstream infections). Infections classified as infective endocarditis correspond to bloodstream infections with infective endocarditis and without any other septic metastasis.

CC398 MSSA was found to be associated with colonization as well as with a wide range of infections (**Figure 1**). Skin and soft tissue infections, bacteraemiae, and pneumoniae were the three most frequent infection types (63/89, i.e. 70.7%). On the basis of a French national prospective study, the prevalence rate of CC398 MSSA within a collection of 132 MSSA strains isolated in 2008 from human infective endocarditis was 7.5% (10/132), thus representing the sixth most frequent CCs after CC45, CC5, CC15, CC30 and CC8. In the same way, upon MSSA strains collected from nasal carriage in a French healthy population, CC398 MSSA was highly prevalent and represented 24.7% of all isolates (21/85).

Erythromycin resistance was the most common within CC398 MSSA (93/105, i.e. 88.6%), whereas resistance to other antibiotics (except penicillin) were detected in less than 6% of isolates (**Table 1**). A low diversity of antibiotic resistance profiles (namely 11) was observed (**Table 2**), but resistance to erythromycin alone, or associated to penicillin was the two most frequent, representing more than 80% of all profiles. The data from DNA microarrays permitted to genetically explain erythromycin resistance (n = 93) only in 7 cases: 3 strains were positive for *erm*A and *erm*C, 2 for *erm*C alone, 1 for *erm*A alone, and 1 for *msrA* (efflux pump). Conversely, the presence of the *erm*T gene, screened using dedicated PCR, explained erythromycin resistance for all of the 86 remaining strains (92.5% of the 105 isolates tested).

**Table 1:** Frequency of antibiotic resistance of 105 MSSA CC398 strains of human origin isolated between 1999 and 2011 in mainland France.

Antibiotics	No. resistant strains (%)n = 105 (100%)
Penicillin	40 (38)
Kanamycin	2 (1.9)
Tobramycin	0
Gentamicin	0
Chloramphenicol	0
Tetracycline	4 (3.8)
Erythromycin	93 (88.6)
Lincomycin	6 (5.7)
Pristinamycin	0
Rifampicin	0
Cotrimoxazole	0
Levofloxacin	0
Fosfomycin	0
Fusidic Acid	2 (1.9)
Linezolid	0

**Table 2:** Frequency of antibiotic resistance profiles of the 105 CC398 MSSA strains of human origin isolated in mainland France.

Antibiotic resistance profile	Number of strainsn = 105 (%)
ERY	55 (52.4)
ERY PEN	29 (27.6)
ERY LIN	1 (0.9)
ERY LIN TET	2 (1.9)
ERY PEN LIN	1 (0.9)
ERY PEN LIN TET	2 (1.9)
ERY PEN KAN	1 (0.9)
ERY PEN FUS	2 (1.9)
PEN KAN	5 (4.8)
KAN	1 (0.9)
All susceptible	6 (5.7)

ERY = erythromycin; PEN = penicillin; LIN = lincomycin; TET = tetracyclin; KAN = kanamycin; FUS = fusidic acid.

DNA microarrays allowed the screening of 172 genes. Analysis of the 35 antibiotic resistance genes (excluding genes of the SSC*mec* cassette) confirmed the multi-susceptible profile of the CC398 MSSA strains: whereas *tet*_Efllux_ spot was constantly positive and *bla*Z/*bla*I/*bla*R operon was detected in 40 out of 105 isolates, only 6 other resistance genes were detected and were distributed only in a few isolates (*erm*C, n = 5; *qac*C, n = 5; *erm*A, n = 4; *tet*M, n = 3; *far*1, n = 2; *msr*A, n = 1). Equipment in specific staphylococcal virulence factors included enterotoxins (*see*, n = 1; *sek* and *seq*, n = 1), Panton-Valentine leukocidin (*luk*S-PV/l*uk*F-PV, n = 6) and TSST-1 (*tst*, n = 1).

The genetic background of the 105 CC398 MSSA isolates was explored using *spa* typing and microarrays, and was found to be highly homogenous. If 25 different *spa*-types were identified, 85% (89/105) of the strains were clustered in a unique *spa*CC (*spa*CC 571) and the phylogenic analysis showed 80% homology of *spa* repeats between all strains (**see Figure S1**). This homogeneous structure of the MSSA CC398 population was confirmed by DNA microarrays data, with constant results for 73.8% of the genes (127 out of 172: 49 always positive and 78 always negative) but also differences (positive versus negative) impacting less than 3% of the 105 isolates, and concerning 27 other genes. The clustered analysis based on microarray data divided the strains into 2 subgroups (**Figure S2**), matching presence/absence of penicillinase (*bla*Z).

The same phenotypic and genetic data was compared to those obtained with CC398 MRSA population collected from colonized animals in France (n = 53 isolates). Unlike human MSSA CC398, CC398 MRSA presented highly diverse multiresistant antibiotic phenotypes, confirmed by the identification of more than 25 different resistance genes profiles. Conversely, a homogenous background was highlighted by *spa* typing; all isolates were distributed in 6 *spa*-types that all belonged to the same *spa*CC571, as CC398 MRSA (**Figure S3**). In the same way, DNA microarrays revealed constant results for more than 68% of the genes within CC398 MRSA collection. The clustered analysis based on microarray data divided the strains into 2 subgroups, related to the acquisition of a different SCC*mec* cassette, namely type IV or type V (**Figure S4**). Finally, it must be noticed that *erm*T gene was absent within this collection and especially in erythromycin resistant strains (unlike their MSSA counterparts).

**Figure 2:**
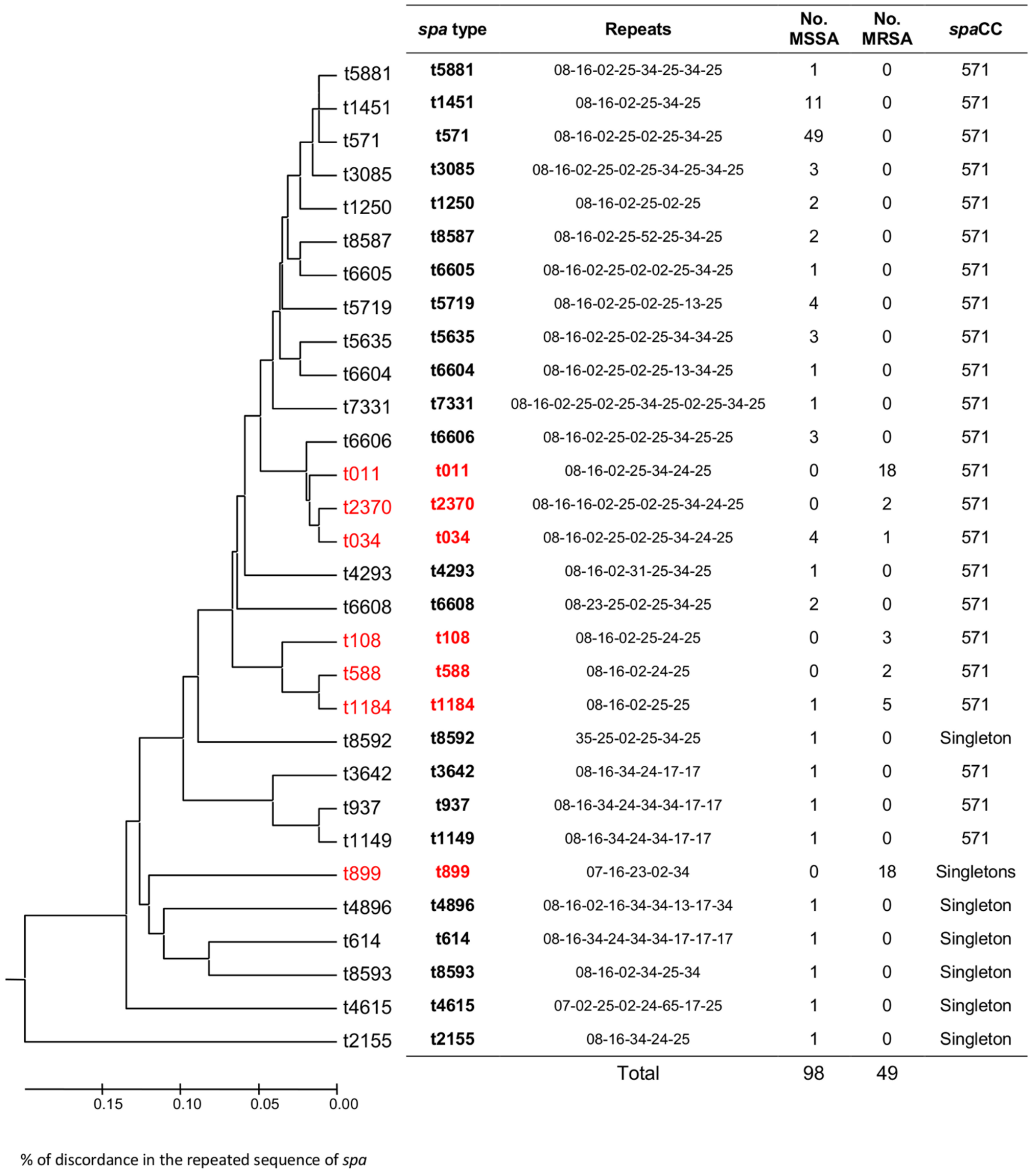
Dendrogram (UPGMA method, squared representation) based on the homology degree of the *spa-types* of the 105 MSSA CC398 strains and the 53 MRSA CC398 isolated in France. The homology between the repeats of the strains is of 76%. *spa*CC: *spa* clonal complex. s*pa types* corresponding to MRSA strains are colored in red.

**Figure 3:**
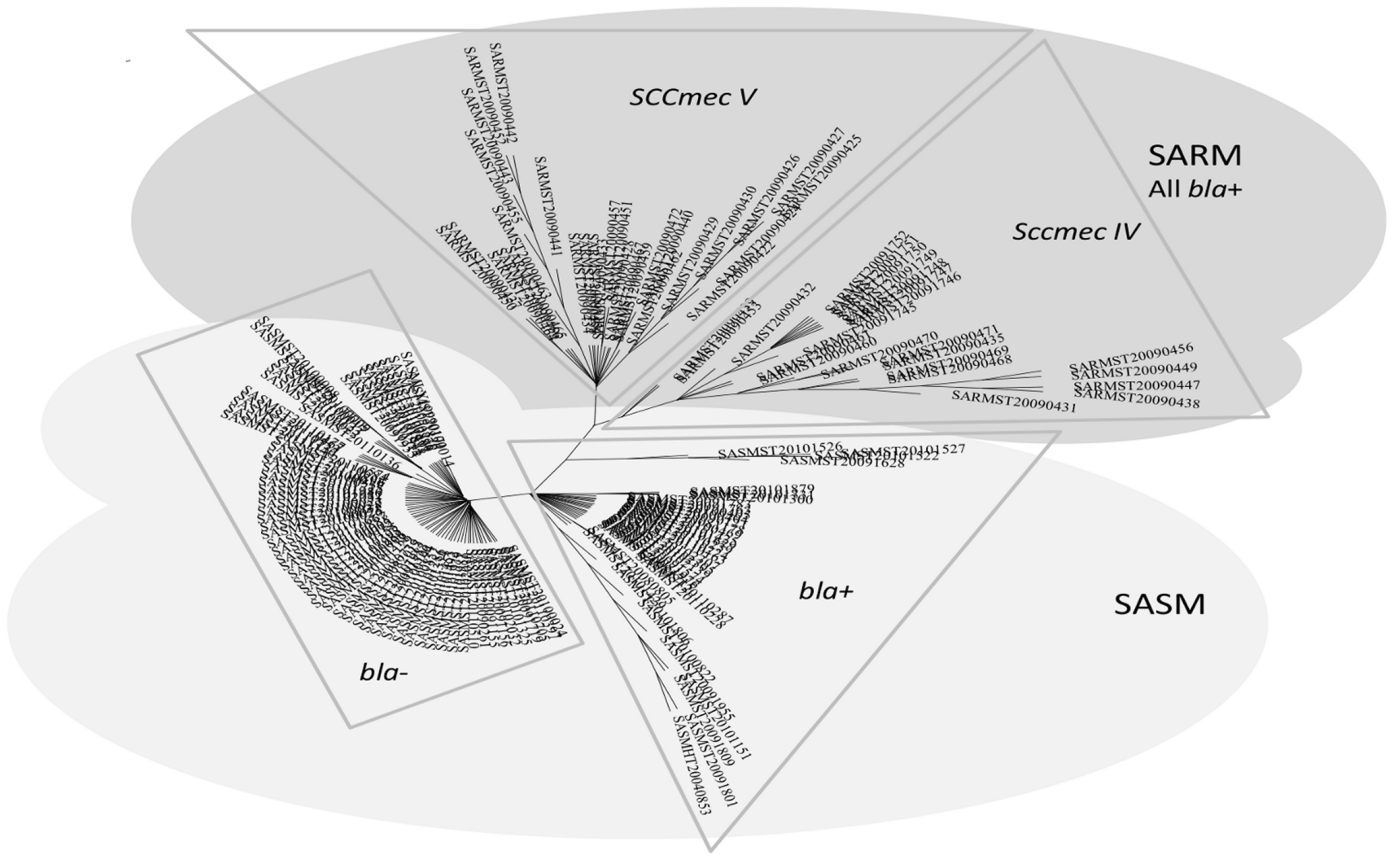
Phylogenic tree with 158 *S. aureus* CC398 strains (modified *Parsimony* method, circular representation), based on the analysis of 319 genes and alleles by DNA microarrays. Since the PCR analysis of the *agr* alleles confirmed that all the strains were *agr* 1, the analysis of the results of DNA microarrays concerned only 319 genes and alleles. MSSA group is subdivided in *bla−* and *bla+* clusters, and MRSA group in SCC*mec* IV and SCC*mec* V clusters.

**Figure 4:**
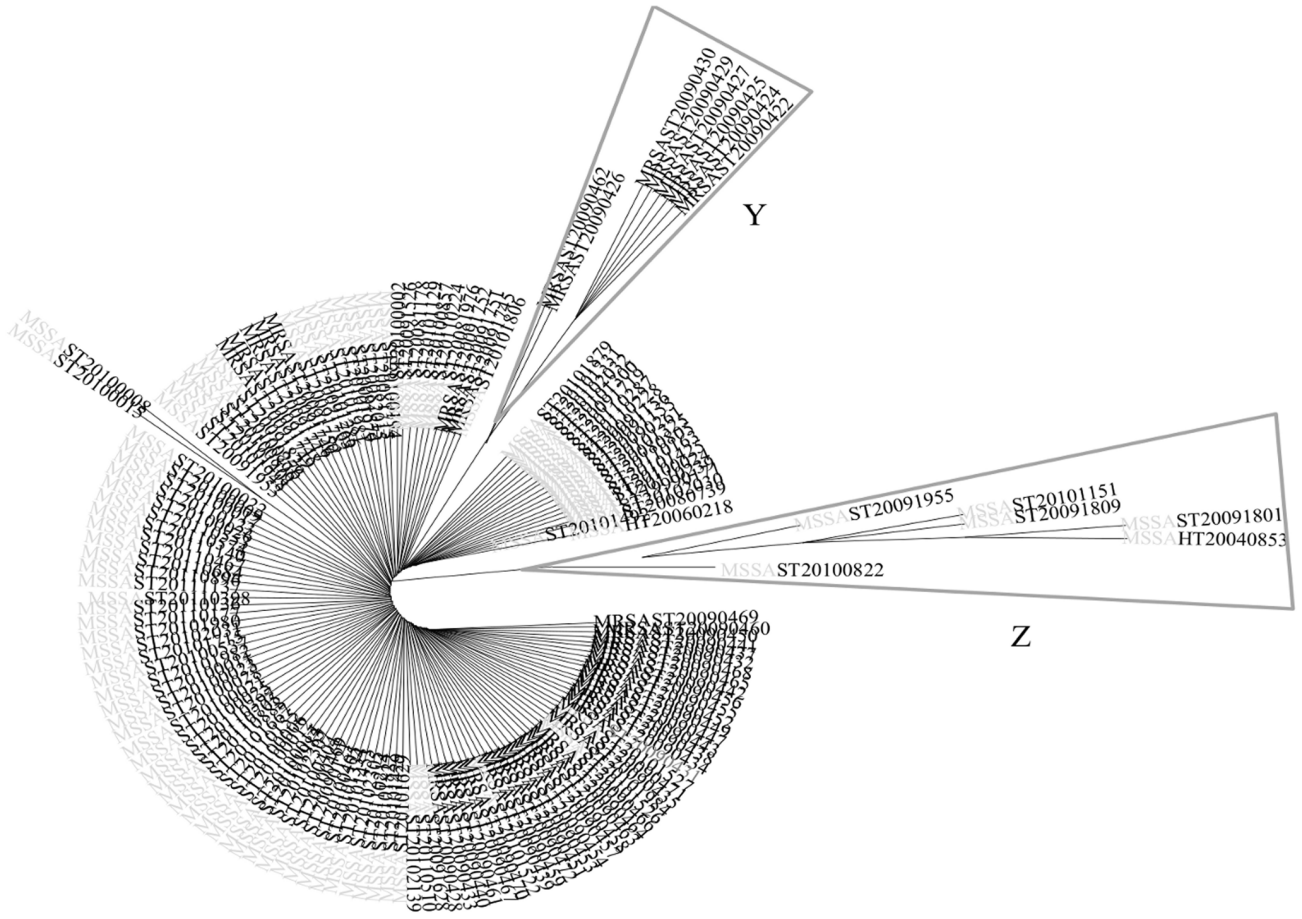
Phylogenic tree with 158 *S. aureus* CC398 strains (modified *Parsimony* method, circular representation), based on the analysis of 274 genes and alleles by DNA microarrays, after exclusion of the 45 antibiotic resistance genes, and the *agr* variants, since the PCR analysis of the *agr* alleles confirmed that all the strains were *agr* 1. Human MSSA are in light grey and animal MRSA in black. The Z branch groups 7 strains presenting a more frequent equipment in virulence genes and alleles than the others (*lukE, sak, splA, splE, ssl07/set1 (MRSA252), hysA2 (All Other Than COL+USA300+NCTC)*), but also 2 hyaluronate lyase genes (*hysA2 (AllOtherThan COL+USA300+NCTC)*). The Y branch gathers 8 strains with some of the virulence genes (*lukS (ST22+ST45), lukX, chp, scn, set6-var4_11, ssl07/set1, ssl11/set2 (MRSA252)*) and one of an adhesine (*clfA (MRSA252)*) less frequent.

The compared analysis of the 105 human CC398 MSSA and the 53 animal CC398 MRSA strains (**Figure 2**) showed a higher diversity of *spa*-types within CC398 MSSA compared to CC398 MRSA (15 versus 6, Simpson diversity index = 88.9% (95%CI, 86.8%–90.9%)). In the same way, DNA microarrays showed that a major part of the constant genes were shared by the two MSSA and MRSA groups (n = 86, i.e. 82.7% of the constant MSSA genes, 73.5% of the constant MRSA genes, and 50% of all the studied genes). The clustered analysis based on microarray data divided the strains into the 4 subgroups described before (**Figure 3**): two subgroups within MSSA matched presence/absence of penicillinase (*bla*Z) and two within MRSA related to the presence of SCC*mec* IV or SCC*mec* V. Interestingly, the exclusion of the 45 antibiotic resistance genes of this analysis lead to a representation with an unique cluster (**Figure 4**). When a global statistical analysis of DNA microarray data was performed, eighteen genes appeared significantly associated with either the livestock CC398 MRSA or the human CC398 MSSA in univariate analysis (**Table 3**). The majority of these genes (16/18) had functions related to antimicrobial or antiseptic resistances and was significantly associated to livestock MRSA isolates, with *tetM* being constant; however, two genes, namely *scn* and *chp*, were significantly associated with the human CC398 MSSA. In both methods (*spa*-typing and DNA microarrays), the corresponding phylogeny made appear MRSA strains as the most derived lineage (**Figures 2** **and** **5**).

**Figure 5:**
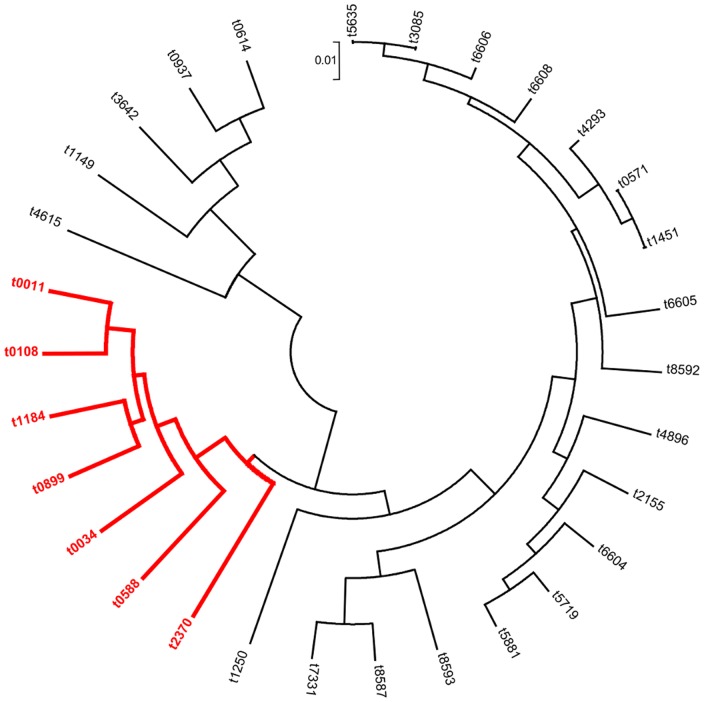
Dendrogram (UPGMA method, circular representation) based on the results of DNA microarrays study (172 genes only) of the 105 MSSA CC398 strains and the 53 MRSA CC398 isolated in France. For each of the 172 genes, the average of the results of all the strains corresponding to the same *spa* type was calculated. Each group of strains is represented by the corresponding *spa* type. s*pa* types corresponding to MRSA strains are colored in red.

**Table 3:** Genes significantly associated with animal and human isolates belonging to the ST398 lineage.

Gene	Protein or function	Livestock MRSA isolates (%)* n = 53	Human MSSA isolates (%)* n = 105	Ambiguous microarray results (%)	P-value**	Odds ratio (CI95)
*tetM*	Tetracycline resistance determinant (ribosomal protection)	52 (100.0)	3 (2.9)	4 (2,5)	1,1128E-35	Inf(247.84-Inf)
*scn*	Staphylococcal complement inhibitor	8 (15.1)	99 (96.1)	2 (1,3)	6,62018E-24	0.01(0.00–0.03)
*chp*	Chemotaxis inhibitory protein	8 (15.1)	100 (95.2)	0 (0,0)	3,13392E-23	0.01(0.00–0.03)
*bla* operon	Beta-lactamase	53 (100.0)	40 (38.1)	0 (0,0)	1,85019E-14	Inf(20.59-Inf)
*dfrA*	Trimethoprim resistance determinant	21 (40.4)	0 (0.0)	2 (1,3)	5,61655E-10	Inf(15.91-Inf)
*tetK*	Tetracycline resistance determinant (active efflux)	13 (33.3)	0 (0.0)	14 (8,9)	1,21203E-06	Inf(11.11-Inf)
*aadD*	Aminoglycosides resistance determinant	15 (28.8)	0 (0.0)	1 (0,6)	2,10074E-06	Inf(9.41-Inf)
*vgaA*	Streptogramin A resistance determinant	15 (28.3)	0 (0.0)	0 (0,0)	2,63481E-06	Inf(9.18-Inf)
*ermC*	Macrolides resistance determinant	19 (35.8)	5 (4.8)	0 (0,0)	0,000128677	10.98(3.61–40.58)
*qacC*	Quaternary ammonium cations resistance determinant	14 (26.4)	5 (4.8)	0 (0,0)	0,025587755	7.08(2.22–26.84)

* For each gene, isolates with ambiguous microarray result were excluded from the analysis and from P-value calculation. For this reason, percentages are not necessarily calculated relative to the total no. of isolates.

** P-values were calculated for each gene using a two-tailed Fisher's exact test and corrected for multiple testing using the Holm-Bonferroni method. Inf, positive infinite.

Noteworthy, the characterization of 26 CC398 MSSA strains collected from human abroad or outside of mainland France showed genetic and phenotypic features matching those observed in isolates from our French collection (data not shown) demonstrating the worldwide representativity of the CC398 MSSA phenomenon described here.

## Discussion

In this study, we described the largest published collection (in number and in period of time) of human CC398 MSSA isolates. Our data highlighted that CC398 MSSA are unspecialized pathogens able to colonize nares as well as to induce a large spectrum of infections. This population of *S. aureus* has specific phenotypic and genotypic signatures including resistance to erythromycin encoded by the *erm*T gene, and expression of staphylococcal complement inhibitor and the chemotaxis inhibitory protein, respectively encoded by *scn* and *chp* genes (major staphylococcal defense/evasion mechanism against the human inanimate immune system) [19]. On the other hand, the CC398 MRSA signature included a panel of antibiotic resistance genes, especially *tetM*. The genetic background of both populations was homogenous, that may come from common ancestress, which may have evolved in the settings of different selective pressures, explaining the acquisition of *ermT*, *chp* and *scn* in the first case, and antibiotic resistance genes in the second.

On the basis of our large strain collection, particular MSSA CC398 characters have been highlighted. Since they do not possess any clone-specific virulence factor, MSSA CC398 strains are able to induce a large spectrum of diseases, unlike “professionalized” *S. aureus* clones (such as TSST-1 producing clones involved in toxic shock syndrome [20] or PVL producing clones, involved in primary skin and soft tissue infection or necrotizing pneumonia). In our study, 89 MSSA strains were involved in 10 different types of infections, even if the 3 more frequent types (skin and soft tissue infections, bacteremia, and pneumonia) represented more than 70% of the overall. The presence of a specific toxin encoding gene appears to be scarce, all the more that a bias of recruitment surely exists in our collection, since FNRCS is recognized for its expertise in staphylococcal toxins, that probably prompts French labs to send to FNRCS isolates involved in apparent human staphylococcal toxinic syndromes. Nevertheless, acquisition of the PVL genes were found to be possible (in only 6 out of 105 MSSA strains), as already reported by Rasigade *et al.* in one case of necrotizing pneumonia [12], but also by Uhlemann et al. in skin infections [21]. On the other hand, the recent description of a prevalence of 64.3% (18/28) for *luk*S-PV and *luk*F-PV in community-acquired ST398 MSSA involved in SSTIs in China demonstrated that the acquisition of such virulence factor and/or spread of such isolates are a source of concern in view of the high fitness of ST398 MSSA for humans. Indeed, epidemiological data collected here revealed a high frequency of CC398 MSSA in MSSA population involved in human colonization and infections. The prevalence rate of CC398 MSSA was 24.7% in a local prospective study on nasal colonization, and reached 7.5% in a national prospective study on infective endocarditis. Such a high prevalence has been reported in some studies elsewhere over the world: in China, where two studies [15], [22] reported ST398 MSSA population as the most frequent ST (18.9% and 17.6% respectively), in West Indies, where it represented 7.8% (7/90) of MSSA or in Dominican Republic and Martinique, where its prevalence reached 10.4% (9/87) [16], but also in Northern Manhattan, in non-invasive infections (5%) [21]. Conversely, in a large-scale European study, Grundmann *et al.* found that MSSA ST398 represented only 1.3% of the 1923 isolates prospectively collected in 26 countries in 2006–2007 [23]: nevertheless, this study included only invasive strains, and mostly involved in bacteremiae, what could emphazise this result. In the same way, Valentin-Domelier *et al.* reported during a prospective French multicentric study that ST398 MSSA represented 2.8% (17/615) of *S. aureus* bloodstream infections (with a growing prevalence between 2007 and 2010) [11]; in agreement with Uhlemann *et al.* reporting that ST398 MSSA was involved in 2.5% of bloodstream infections in Northern Manhattan [21].

Another interesting feature of MSSA CC398 is its frequent exclusive erythromycin resistant phenotype, encoded by the *erm*T gene: 86 of the 93 erythromycin-resistant MSSA CC398 strains were positive using a specific PCR. This gene has initially been detected in lactobacilli, streptococci and enterococci [24], and has recently been found in MRSA ST398 of animal origin, carried by a plasmid [24]–[26] (with a perfect sequence homology with the *erm*T gene isolated from *Streptococcus pyogenes* [24], [25]); but also in MSSA ST398 strains isolated from humans in Spain, Belgium, and Northern Manhattan [10], [14], [21]. Vandendrissche *et al.*, studying the restriction pattern of 5 MSSA CC398 isolates showed that *erm*T was integrated in the chromosome [14]. Intriguingly, in our collection of 53 animal CC398 MRSA strains, *erm*T was completely absent, unlike other erythromycin-resistance conferring genes. Since the antibiotic selective pressure may be present in both cases, this difference might traduce a different microbiological environment between human CC398 MSSA and animal CC398 MRSA (and thus different horizontal transfer possibilities), or different genetic (or epigenetic) backgrounds in these two groups (and thus different integration capacities for the same resistance genes).

The close genetic background observed for the two MSSA and MRSA CC398 populations using microarray and *spa*-typing combined with the presence of human virulence factors (toxins, *scn*, *chp*) but poverty of antimicrobial resistance gene profiles (except *erm*T) in MSSA, and with the absence of specific human virulence factors but a high diversity of antimicrobial resistance gene profiles in MRSA, is suggestive of a different and independent evolutive history of the two populations. The level of diversity and segregation of *spa*-type and the specific gene content observed for the two populations strongly suggest that CC398 lineage originated in humans and then has undergone a radiation driven by the jump from humans to animals. The change of niches would have induced the loss of useless human virulence factors (such as *scn* or *chp*, that have no impact and confer no advantage in animals), and the acquisition of some others, such as various antibiotics resistance genes, including tetracyclin and methicillin resistance in connection to the strong and specific antimicrobial selection associated with livestock production. Recently, results of whole–genome sequencing approaches, strongly reinforced by the fact that CC398 MRSA showed decreased ability for human colonization transmission and virulence, support this point of view [21], [27]–[29]. Nevertheless, the lack of some “missing links”(like for instance the unexplained different distribution of the *erm*T gene between MSSA and MRSA CC398 populations) could suggest another evolutive history. An external and undescribed third MSSA ST398 reservoir would have alternatively been at the source of the human niche and the animal niche, with respective specific selective adaptation to host and antibiotic pressure. Genetic recombination likely involving genes from coagulase-negative staphyphylococci or other *S. aureus* clones that beforehand colonized each of these niches, might have played a pivotal role.

Whole-genome analysis has been applied to the study of CC398 MSSA and MRSA and data published recently by Price *et al.* [27] and Uhlemann *et al.* [21]. Such approaches are relevant for studying genetic content and diversity among populations but cannot be applied to routine analysis, since they are expensive, and require bioinformatic allocated resources and time-consuming analysis. Although microarrays cover only a part of the *S. aureus* genome, we showed that data matched those obtained by whole-genome approaches and are easily obtained. Thus, microarrays allow the rapid and cheap identification of animal or human origin of CC398 isolates. A detailed analysis of data shows, that a multiplex PCR targetting *scn*, *chp*, *erm*T could be useful for simply and efficiently discriminate animal and human origin of such isolates.

Because of a retrospective design, this work presents selection bias. Indeed, the strains described here were mainly collected passively by the CNRS, or within the framework of research projects: consequently, they may not be as representative of the global MSSA CC398 population as would be strains prospectively and randomly selected. Nevertheless, we described the tendency that CC398 MSSA strains might present in terms of epidemiology and characteristics.

On the basis of this work, we assume that MSSA CC398 is a frequent cause of human colonization and infections, suggesting a well-adapted fitness of this clone to humans. This contrasts with the behavior of CC398 MRSA, which seems to be more adapted to animal than to human beings [30], [31]. We detected no patent differences in terms of adherence factors between these two populations, but found a more frequent distribution of *scn* and *chp* (but not *sak* neither *entA* that are often clustered on the same phages) in the MSSA group. These two genes are parts of the immune evasion cluster located on the conserved 3′ end of beta-hemolysin converting bacteriophages, easily horizontally transferable between different *S. aureus* strains [19]. They have already been shown to be associated to *S. aureus* human host specificity for the ST398 lineage [11], [21], [27], [32], but not only [33]. Since the microarrays covered a small part of the *S. aureus* genome and were only based on small probes designed to target conserved genetic regions, their use gives us only a partial view of the gene content and the gene polymorphism. Whole genome sequencing approach would allow us to open new possible lines of reflection. Besides, Uhleman et al., using preliminary comparison of three fully sequenced ST398 MSSA and MRSA genomes, identified mutations and deletions in genes encoding cell surface-bound proteins [21]. These differences must be now correlated with phenotypic features, confirmed by the construction of isogenic couples to demonstrate causality and clinical relevancy. Moreover, the study of the CC398 *S. aureus* at the phenotypic or regulation/dysregulation system levels could give us other keys.

## Materials and Methods

One hundred and five CC398 MSSA human isolates were included from the collection of the French National Center for Staphylococci (**Table 4**). This collection included isolates voluntarily sent by French microbiology laboratories for further characterization because of particular antibiotic resistance phenotype or unusual infections, or in the setting of targeted studies. Thus, between March 2009 and May 2011, DNA microarrays (Identibac *S. aureus* Genotyping ®, Alere) [25], [34], used in routine, allowed the prospective identification of 94 isolates as members of CC398 on the basis of clonal assignment provided by the software of the kit. Eleven additional strains were included retrospectively after being tested using the same DNA microarrays for various purposes. Eighty-nine isolates were involved in infections and 11 in colonization (5 unknown). Standardized clinical and demographic information was collected for all CC398 MSSA strains that caused infections (n = 89): age and gender of each patient, infection type, date and site of isolation. In addition, to evaluate the prevalence of CC398 MSSA in specific populations, a collection of 85 MSSA isolated from human healthy carriers (nasal colonization) and a collection of 132 MSSA collected during a French national prospective study of human infective endocarditis (infections diagnosed in 2008) were screened for CC398. Fifty-three CC398 MRSA strains from animal origin were used as comparators. Finally, 26 CC398 MSSA strains collected from human abroad or outside of mainland France (Switzerland, n = 6; China, n = 5; US, n = 3; Algeria, n = 2; Denmark, n = 3; Martinique, n = 2; Dominican Republic, n = 2; India, n = 1; Madagascar, n = 1; La Reunion island, n = 1; French Guyana, n = 1) were included and characterized.

**Table 4:** Description of the studied populations, corresponding used methods, and objectives.

Studied populations	Of which	Of which	Of which	Used methods	Objectives
**158** retrospectively gathered CC398 *S. aureus* strains	**105** human MSSA strains	**105** strains isolated in mainland France	**89** strains from infections11 from colonization5 unknown	Clinical informationAntimicrobial susceptibility*spa* typingDNA microarraysPCR CC398†	Phenotypic and genotypic characterization of CC398 MSSA isolated from human beings, and comparison to CC398 MRSA isolated from animals
	**53** animal MRSA strains	45 strains from pigs8 from cattle	Only colonization		
**85** prospectively isolated MSSA strains of human colonization	**21** CC398 MSSA strains			*agr* typingPCR CC398	Estimation of CC398 MSSA prevalence
**132** prospectively isolated MSSA strains of human infective endocarditis	**10** CC398 MSSA strains*			*spa* typingDNA microarraysPCR CC398	Estimation of CC398 MSSA prevalence

* The 10 strains isolated from infective endocarditis are included in the 89 strains from infections.

† The belonging of the strains to CC398 was confirmed by MLST in case of discrepancies between previous typing techniques. All strains were isolated in mainland France.

Susceptibility to 16 antibiotic drugs was determined with the standard agar diffusion technique, as recommended by the French Society for Microbiology [35]. Genomic DNA was extracted using a standard procedure on Qiacube [20]. *agr* alleles and the presence of *mec*A gene were determined by specific PCR [20]. *erm*T gene was detected using specific primers and amplification protocol based on the description by Kadlec *et al.* [25], [36], with modifications (see the **Appendix**). *spa*-typing was performed with the Ridom Staph Type standard protocol [37] and by using *Ridom Staph Type software®* (version 1.5) [38] (http://spaserver.ridom.de/), which automatically analyses *spa* repeats and assigns *spa* types. *spa* types were clustered into clonal complexes CCs (ie, *spa*CCs) using the integrated BURP (Based Upon Repeat Patterns) algorithm [37]. *spa* phylogenic trees were constructed based on this analysis using the UPGMA reconstruction algorithm. DNA microarray analyses (Identibac *S. aureus* Genotyping ®, Alere) were performed according to the protocol described by Monecke et al. [34], in aim to characterize the *S. aureus* isolates. These microarrays detected 330 target sequences corresponding to 172 genes and their allelic variants. The data were interpreted based on the algorithm previously described [34]. A split network tree was generated using the modified Parcimony method and the SeaView program version 4 [39]. Distributions of the 172 genes were compared between the MSSA and MRSA groups. Univariate statistical analysis was performed using a two-tailed Fisher's exact test. *p*-values were corrected for multiple testing using the Holm-Bonferroni method. For each gene, isolates exhibiting ambiguous microarray results were removed from the analysis. The statistical significance threshold for all tests was set to 0.05. Because of the close genetic relationships between isolates, strong colinearity occurred in gene distributions; multivariate analysis was thus deemed irrelevant and was not performed. All statistical analyses were performed by means of the R software version 14.0 (The R Foundation for Statistical Computing, Vienna, Austria).

## Appendix

***ermT*** **gene specific PCR protocol**. The *ermT* gene was detected using specific primers and amplification protocol based on the description by Kadlec et al. [25], [36], but with modifications. For each probe, the mix was composed of 1,5 UI of Taq-polymerase *Eurobio®*, 5 µl of reaction buffer *Eurobio®* without MgCl2 (10×), 2 µl of MgCl2 *Eurobio®* (50 mM), 5 µl of dNTP (20 µM), 5 µl of each one of the 2 primers (2 pmol/µl), and 5 µl of DNA. The amplification stage was composed of 30 cycles, with a phase of denaturation of 30 sec at 94°C, of hybridation of 30 sec at 57°C, and elongation of 40 sec at 72°C. Amplification products were obtained at the expected size; one of them was sequenced, and the result was blasted with a 99% homology with the reference *ermT* gene found in GenBank (http://www.ncbi.nlm.nih.gov/blast/Blast.cgi).

## Supporting Information

Figure S1 **Dendrogram (UPGMA method, squared representation) based on the homology degree of the** ***spa-types*** **of the 105 MSSA CC398 strains of human origin isolated in France.** The homology between the *spa* repeats is of 80%. Of note, 6 other strains (6%) corresponding to 3 *spa* types (t3625 (n = 3), t4316 (n = 1), t8698 (n = 2)) were excluded from the analysis since it had a repeated sequence shorter than 5 repeats. And 1 strain were non typable by *spa* typing.
(TIF)

Figure S2 **Phylogenic tree with 105 MSSA CC398 strains (modified** ***Parsimony*** **method, circular representation), based on the analysis of 319 genes and alleles by DNA microarrays.** Since the PCR analysis of the *agr* alleles confirmed that all the strains were *agr* 1, the analysis of the results of DNA microarrays concerned only 319 genes and alleles. *bla*-negative strains are grouped in the *bla−* cluster (light grey), *bla*-positive strains in the *bla+* cluster (dark grey). (n: number of strains) The Z branch is composed of strains more frequently positive for some virulence factors. The green strain is positive for *tsst1*; the one in red for *pvl*; these over lined in blue for *edinB*; and these underlined for *etd*. Within the *bla*+ population 7 strains are isolated in a branch Z, presenting a more frequent equipment in virulence genes and alleles than the others (*lukE, sak, splA, splE, ssl07/set1 (MRSA252), hysA2 (All Other Than COL+USA300+NCTC)*), but also 2 hyaluronate lyase genes (*hysA2 (AllOtherThan COL+USA300+NCTC)*).
(TIF)

Figure S3 **Dendrogram (UPGMA method, squared representation) based on the homology degree of the** ***spa-types*** **of the 53 MRSA CC398 strains of animal origin isolated in France.** The homology between the *spa* repeats is of 86%. Of note, the *spa* type t1456, gathering 4 strains, was excluded of the analysis, having a too short repeated sequence. *spa*CC: *spa* clonal complex.
(TIF)

Figure S4 **Phylogenic tree with 53 MRSA CC398 strains (modified** ***Parsimony*** **method, circular representation), based on the analysis of 319 genes and alleles by DNA microarrays.** The strains carrying the type IV *SCCmec* cassette are grouped in a first cluster (light grey), and strains carrying the type V *SCCmec* cassette in a second one (dark grey). The 8 strains of cattle origin are separated from the others. Since the PCR analysis of the *agr* alleles confirmed that all the strains were *agr* 1, the analysis of the results of DNA microarrays concerned only 319 genes and alleles.
(TIF)
