# Supplementary figures and images for: Clonal Complex 398 Methicillin Susceptible Staphylococcus aureus: A Frequent Unspecialized Human Pathogen with Specific Phenotypic and Genotypic Characteristics
Source: PLoS One. 2013 Nov 15;8(11):e68462. doi: 10.1371/journal.pone.0068462 (PMC3829818; doi:10.1371/journal.pone.0068462)

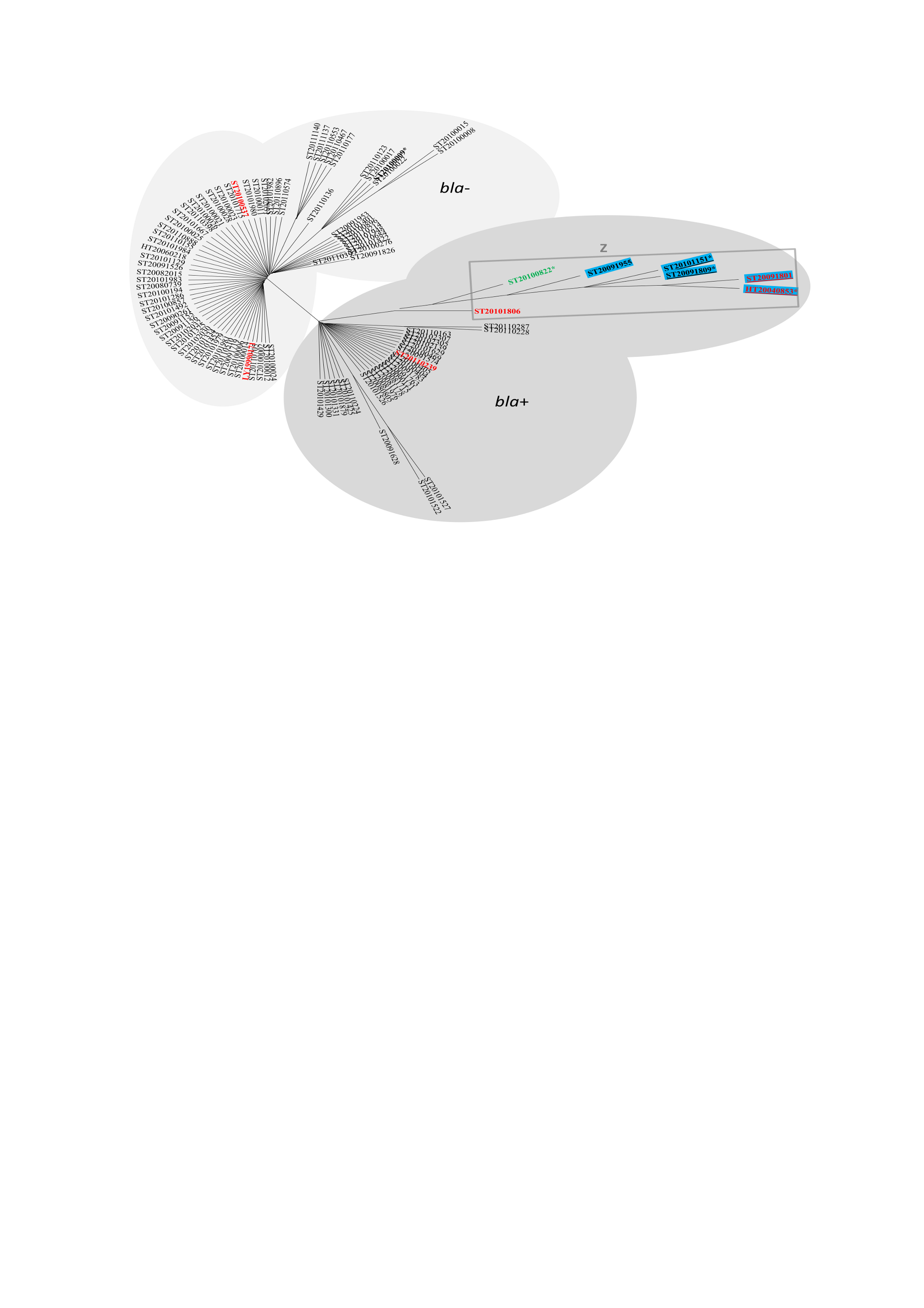

Supplement: Figure S2 — Phylogenic tree with 105 MSSA CC398 strains (modified Parsimony method, circular representation), based on the analysis of 319 genes and alleles by DNA microarrays. Since the PCR analysis of the agr alleles confirmed that all the strains were agr 1, the analysis of the results of DNA microarrays concerned only 319 genes and alleles. bla-negative strains are grouped in the bla− cluster (light grey), bla-positive strains in the bla+ cluster (dark grey). (n: number of strains) The Z branch is composed of strains more frequently positive for some virulence factors. The green strain is positive for tsst1; the one in red for pvl; these over lined in blue for edinB; and these underlined for etd. Within the bla+ population 7 strains are isolated in a branch Z, presenting a more frequent equipment in virulence genes and alleles than the others (lukE, sak, splA, splE, ssl07/set1 (MRSA252), hysA2 (All Other Than COL+USA300+NCTC)), but also 2 hyaluronate lyase genes (hysA2 (AllOtherThan COL+USA300+NCTC)). (TIF) [file pone.0068462.s002.tif]
